# Supplementary material for: Irritability in young people with copy number variants associated with neurodevelopmental disorders (ND-CNVs)
Source: Transl Psychiatry. 2024 Jun 18;14:259. doi: 10.1038/s41398-024-02975-z (PMC11189457; doi:10.1038/s41398-024-02975-z)
Supplement: Supplementary file 1 — Supplementary Figure Legends [file 41398_2024_2975_MOESM1_ESM.docx]

**Supplementary Figure Legends:**

**supplementary table 1: description of number of participants with each genotype within sample**

**supplementary table 2: psychiatric and epilepsy medications being used in sample. adderall, atomoxetine, methylphenidate, are typically prescribed to treat adhd. carbamazepine, lamotrigine, sodium valproate and topiramate are typically prescribed to treat epilepsy. risperidone and aripiprazole are antipsychotic medications which may have also been prescribed “off label” to treat adhd, autism, obsessive compulsive disorder, depression, behavioural disorders, tics or tourettes. lithium is used to treat bipolar disorder. sertraline and fluoxetine are selective serotonin reuptake inhibitors typically used to treat depression and anxiety.**

**Supplementary table 3: number and proportion of nd-cnv carriers and controls who met criteria for each of the symptoms in the irritability construct; touchy or easily annoyed, angry and resentful, and temper tantrums. To meet criteria for each symptom, the symptom had to be present at least 3-4 times per week in the last three months. Table also presents the number and proportion of individuals who met for at least 1, 2 or 3 of the symptoms from the irritability construct.**

**supplementary table 4: results from mixed effects logistic regression models with adhd, anxiety, indicative autism, mood disorders and “any psychiatric diagnosis” as the outcome variables with nd-cnv as the predictor variable, controlling for irritability, age and gender. Nd-cnv status and irritability are associated with adhd, anxiety, indicative autism and any psychiatric diagnosis, but not mood. This demonstrates that the association of nd-cnvs with psychiatric disorders is, at least in part, independent of the association between nd-cnvs and irritability.**

**Supplementary table 5: results from logistic regression in nd-cnv carriers, with each of the three irritable symptoms as the outcome variable, and fsiq as the predictor, controlling for age and gender. There is no association between fsiq and each of the irritable symptoms in nd-cnv carriers.**
